# 2396: Utility of the Modified Barium Swallow Impairment Profile as an outcome measure in oculopharyngeal muscular dystrophy

**DOI:** 10.1017/cts.2017.135

**Published:** 2018-05-10

**Authors:** Sarah Youssof, Carol Romero-Clark

**Affiliations:** Clinical and Translational Science Center, University of New Mexico, Albuquerque, NM, USA

## Abstract

OBJECTIVES/SPECIFIC AIMS: Oculopharyngeal muscular dystrophy (OPMD) is a rare, late-onset muscular dystrophy that causes severe swallowing impairment (dysphagia). Although promising therapies are in the pipeline, validated dysphagia outcome measures for use in OPMD trials have not been established. Videofluoroscopic swallow studies (VFSS) are considered the clinical gold standard for dysphagia assessment, yet the optimal objective measure of VFSS in OPMD is not known. Our aim was to investigate the utility of the Modified Barium Swallow Impairment Profile (MBSImP) as an objective measure of VFSS in OPMD patients. METHODS/STUDY POPULATION: This was a single-center, prospective, cross-sectional study. In total, 26 individuals with OPMD underwent VFSS and other measures of dysphagia including 50-mL water swallow time (ST). Validity was assessed by examining correlations with an OPMD Global Severity Score (GSS) and with dysphagia duration. RESULTS/ANTICIPATED RESULTS: The MBSImP demonstrated moderate correlations with GSS (Pearson *r*=0.52, *p*=0.006) and ST (*r*=0.39, *p*=0.049). The relationship between MBSImP and dysphagia duration appeared nonlinear, and levelled off with long dysphagia duration. In contrast, ST did not correlate significantly with GSS (*r*=0.27, *p*=0.18), nor with disease duration (*r*=0.05, *p*=0.83). DISCUSSION/SIGNIFICANCE OF IMPACT: Objective measurement of VFSS is a promising outcome measure in OPMD. With long disease duration, the MBSImP may not be sufficiently sensitive to detect disease progression. More sensitive measures for scoring dysphagia severity on VFSS should be explored for application to future s of OPMD.

